# Cultivation-dependent and cultivation-independent characterization of hydrocarbon-degrading bacteria in Guaymas Basin sediments

**DOI:** 10.3389/fmicb.2015.00695

**Published:** 2015-07-07

**Authors:** Tony Gutierrez, Jennifer F. Biddle, Andreas Teske, Michael D. Aitken

**Affiliations:** ^1^Department of Environmental Sciences and Engineering, Gillings School of Global Public Health, University of North Carolina at Chapel Hill, Chapel Hill, NCUSA; ^2^School of Life Sciences, Heriot-Watt University, EdinburghUK; ^3^College of Earth, Ocean, and Environment, University of Delaware, Lewes, DEUSA; ^4^Department of Marine Sciences, University of North Carolina at Chapel Hill, Chapel Hill, NCUSA

**Keywords:** Guaymas Basin, hydrocarbon degradation, stable isotope probing, polycyclic aromatic hydrocarbons (PAHs), *Cycloclasticus*, deep-sea, marine environment

## Abstract

Marine hydrocarbon-degrading bacteria perform a fundamental role in the biodegradation of crude oil and its petrochemical derivatives in coastal and open ocean environments. However, there is a paucity of knowledge on the diversity and function of these organisms in deep-sea sediment. Here we used stable-isotope probing (SIP), a valuable tool to link the phylogeny and function of targeted microbial groups, to investigate polycyclic aromatic hydrocarbon (PAH)-degrading bacteria under aerobic conditions in sediments from Guaymas Basin with uniformly labeled [^13^C]-phenanthrene (PHE). The dominant sequences in clone libraries constructed from ^13^C-enriched bacterial DNA (from PHE enrichments) were identified to belong to the genus *Cycloclasticus*. We used quantitative PCR primers targeting the 16S rRNA gene of the SIP-identified *Cycloclasticus* to determine their abundance in sediment incubations amended with unlabeled PHE and showed substantial increases in gene abundance during the experiments. We also isolated a strain, BG-2, representing the SIP-identified *Cycloclasticus* sequence (99.9% 16S rRNA gene sequence identity), and used this strain to provide direct evidence of PHE degradation and mineralization. In addition, we isolated *Halomonas*, *Thalassospira*, and *Lutibacterium* sp. with demonstrable PHE-degrading capacity from Guaymas Basin sediment. This study demonstrates the value of coupling SIP with cultivation methods to identify and expand on the known diversity of PAH-degrading bacteria in the deep-sea.

## Introduction

In deep-sea naturally oil-laden marine sediments, such as cold seeps, hydrocarbon-degrading microorganisms contribute importantly to the diagenesis, and biological transformation of hydrocarbons. Since microorganisms, in particular oil-degrading bacteria, are the foundation of natural bioremediation processes and protagonists in the removal of hydrocarbon contaminants (Head et al., 2006; Yakimov et al., 2007), identifying these types of bacteria in the deep-sea is a first step to understanding their role in the mineralization of hydrocarbons in these environments and how they would respond to oil from the overlying water column. As evidenced during the Deepwater Horizon blowout, oil in surface waters of the Gulf of Mexico reached the seafloor through the formation and subsequent vertical sedimentation of marine oil snow (MOS; Chanton et al., 2015), which is oil-enriched mucilaginous particulates that had formed within 1–2 weeks of the blowout (Passow et al., 2012). This process can introduce large quantities of oil to the seafloor where it can have acute and lasting impacts to benthic ecosystems. Microbial degradation of hydrocarbons in deep petroleum reservoirs is well-documented (Orphan et al., 2000; Aitken et al., 2004; Jones et al., 2008; Bennett et al., 2013), and hydrocarbon-degrading bacteria in coastal environments have been investigated extensively (Yakimov et al., 2007). However, the deep-sea has received considerably less attention in this respect since the first published reports on hydrocarbon biodegradation in the deep-sea in the 1970s (Schwarz et al., 1974a,b). With exploration and production for oil in deeper water provinces having accelerated in recent years, there is a need to improve our understanding of the diversity and catabolic potential of oil-degrading bacteria in deep-sea sediments.

A model system for studying the diversity and evolution of hydrocarbon-degrading bacteria in the deep-sea exists in the Guaymas Basin (Teske et al., 2014). Located at approximately 2000 m water depth on the seabed in the Gulf of California, this submarine spreading center is characterized by hydrocarbon seeps, hydrothermal plumes, and hot springs. The high temperatures (up to 200–300°C) in sub-surface Guaymas sediments lead to the pyrolysis of organic material in these organic-rich sediments (3–12% [wt/wt] near the sediment surface; Lanza-Espino and Soto, 1999), as well as to the production of significant quantities of petroleum hydrocarbons in the deep subsurface sediments (Peter et al., 1991). The vent fluids, which are laden with petrochemicals, migrate upward to the sediment surface, thus providing a natural model system for studying the microbiology of deep-sea hydrocarbon-degrading communities. These communities are largely responsible for recycling of the hydrocarbons in highly active sediments at Guaymas (Pearson et al., 2005).

Stable-isotope probing (SIP) has been used successfully on environmental samples to identify a microbial group(s) of interest based on their ability to assimilate the stable isotope, thereby being able to link the phylogenetic identity of an organism to its function (Dumont and Murrell, 2005). The technique therefore has promise to explore the diversity of hydrocarbon-degrading bacteria in natural environments, and to link this phenotype to phylogenetic identity. Few reports have, however, applied SIP with hydrocarbon substrates to the study of hydrocarbon-degraders in ocean systems (Gutierrez et al., 2011, 2013b; Mishamandani et al., 2014; Sauret et al., 2014). Recently, the application of SIP has uncovered sulfate-reducing bacteria of the *Desulfosarcina/Desulfococcus* clade as key players in the anaerobic degradation of alkane hydrocarbons at deep marine seeps, including the Guaymas Basin (Kleindienst et al., 2014). Here, we studied PAH-degrading bacteria in sediment cores collected from the Guaymas Basin – a model system for studying the diversity of these organisms in the deep-sea (Teske et al., 2014) and where aromatic/naphthenic hydrocarbon levels (including PAHs) can constitute up to 30% of the oil (Bazylinski et al., 1988). For this, DNA-SIP and cultivation-based methods were used to identify PAH-degrading bacteria in the surficial sediment environment of Guaymas at ~2000 m depth below the sea surface in order to expand current knowledge on the diversity of hydrocarbon-degrading microbial communities in Guaymas Basin oil-rich sediments.

## Materials and Methods

### Field Samples

**FIGURE 1:**
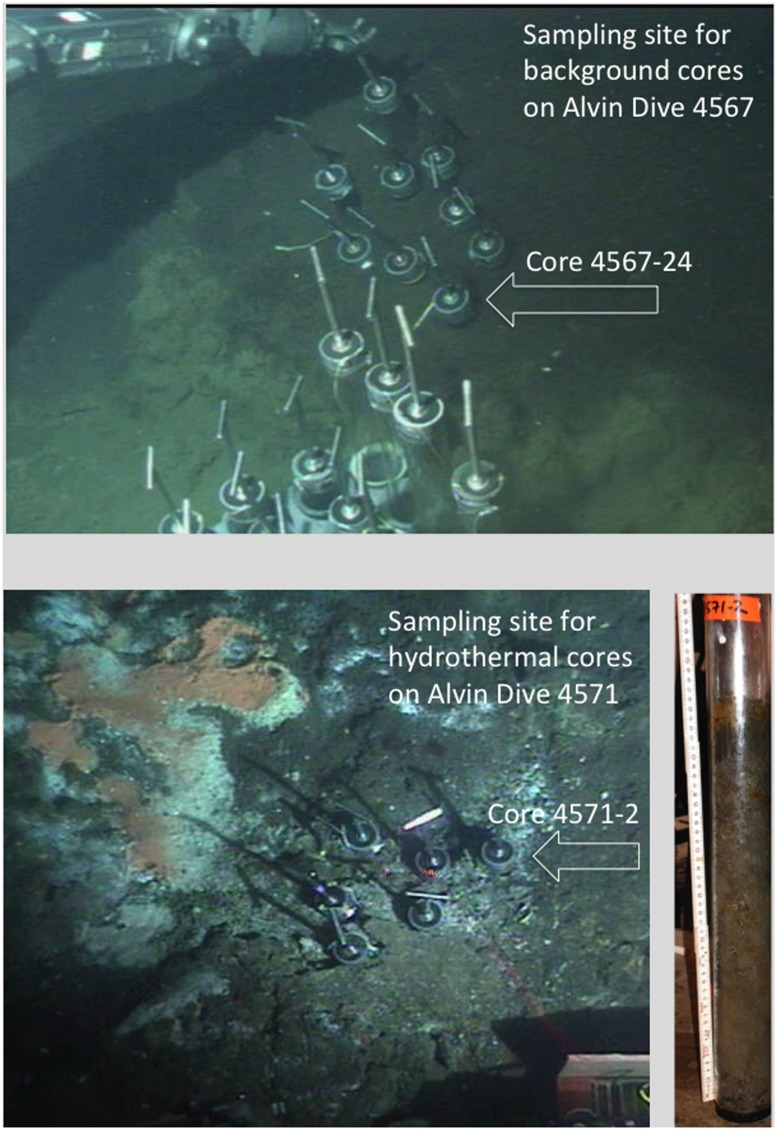
***In situ* still photographs of sampling sites during Alvin dives 4567 and 4571, obtained with the Alvin frame-grabber system (http://4dgeo.whoi.edu/alvin).** The **(Top)** image shows the benthic sediment without microbial mats or hydrothermal features sampled during dive 4567; the **(Bottom)** image shows the microbial mats and sulfur precipitates on the sediment surface that reveal hydrothermal influence. Right, photo of core 4571-2 after shipboard retrieval; the reddish spots are oil droplets in the sediment core.

Samples were collected in 2009 on R/V *Atlantis* cruise AT15-56 by push coring with the submersible *Alvin*. Core 4567-24 was collected on November 28, 2009 from cold non-hydrothermal sediment with an *in situ* temperature of +4 to 5°C throughout the sediment core, no free sulfide, and no overlying bacterial mat, at a water depth of 2011 m at 27°0.542′N, 111°24.488′W. Core 4571-2 was collected on December 2, 2009 from a site with oil-rich sediments next to a well-developed *Beggiatoa* mat, at a water depth of 2007 m at 27°0.388′N, 111°24.560′W (**Figure 1**). This core was characterized by high porewater sulfide concentrations in the range of 2–4.5 mM, and an *in situ* temperature gradient of ca. 10°C at the surface to near 50°C at 40 cm depth, as measured with Alvin’s Heatflow probe (McKay et al., 2012). Cores were brought to the surface, immediately transferred and kept in a cold room +4°C, then sectioned by depth. At the time of collection, aliquots of the core samples 0–4 cm below seafloor were stored in sterile Falcon tubes and kept at +4°C for subsequent use within 2 weeks in enrichment, mineralization, degradation, and SIP experiments (described below).

### SIP Incubations

Prior to commencing SIP incubations, mineralization assays using ^14^C-labeled naphthalene (NAP), phenanthrene (PHE), anthracene (ANT), pyrene (PYR), fluoranthene (FLU), and benz[a]anthracene (BaA) were performed on the collected sediment samples to determine which of these hydrocarbons would warrant SIP with their ^13^C-labeled counterparts. [9-^14^C]-PHE (8.3 mCi mmol^-1^), [1, 2, 3, 4, 4a, 9a-^14^C]-ANT (17.3 mCi mmol^-1^), [4, 5, 9, 10-^14^C]-PYR (61 mCi mmol^-1^), [3-^14^C]-FLU (45 mCi mmol^-1^), and [U-^14^C]-NAP (17.8 mCi mmol^-1^) were from Sigma–Aldrich (St Louis, MO, USA). [5, 6-^14^C]-BaA (54.6 mCi mmol^-1^) was obtained from Chemsyn Science Laboratories (Lenexa, KS, USA). Mineralization assays were conducted in sterile 40-ml amber-glass EPA vials, each containing a ^14^C-labeled test compound (to 20,000 d.p.m., except for PYR which was added to 3,000 d.p.m.) and 2.5 mg of the respective unlabeled test compound in 4.5 ml of ONR7a medium (Dyksterhouse et al., 1995). For the inoculum, 0.5 g of wet sediment sample was inoculated into the vials. Killed controls were prepared by adding 85% phosphoric acid to pH of ≤1 prior to inoculation. All treatments were conducted in triplicate. For the CO_2_ trap, a sterile glass test tube (12 mm × 75 mm) containing a piece of filter paper saturated with 60 ml of 2 M KOH was inserted into each vial. The vials were sealed with foil-covered Teflon-lined caps and incubated with shaking (100 r.p.m.) at 4°C or 21°C in order to determine which incubation temperature would be most suitable for SIP. The filter paper from each vial was removed daily and the captured ^14^C from any ^14^CO_2_ respired was counted on a Packard (Meriden, CT, USA) Tri-Carb liquid scintillation analyzer (model 1900TR). The KOH-saturated filter paper from each vial was replaced at each sampling point for the course of the experiment. The percentage of ^14^C mineralized for each compound was calculated by subtracting the triplicate values for the acidified controls from those of the experimental and then dividing by the total d.p.m. of ^14^C added.

Stable-isotope probing incubations were performed using 125-ml sterilized glass screw-top Erlenmeyer flasks with caps that were lined with aluminum foil to prevent sorption of hydrocarbons. Each flask contained 18 ml of ONR7a medium and 2 g of sediment slurry from core 4567-24. [U-^13^C]-PHE was synthesized by methods described elsewhere (Zhang et al., 2011). Five sets of replicate flasks were prepared and run in parallel. Duplicate flasks were prepared with 1 mg of [U-^13^C]-PHE for the SIP incubation, and a second set of duplicates was prepared with 1 mg of unlabeled PHE. To determine the endpoint of each SIP experiment, the mineralization of [U-^14^C]-PHE was measured in triplicate flasks by liquid scintillation counting of ^14^CO_2_ trapped in KOH-soaked filter paper over time, as described above. An additional set of triplicate flasks was used to monitor the disappearance of the unlabeled PHE by HPLC; samples were periodically taken from these flasks for DNA extraction and subsequent measurement of the abundance of target organisms identified through SIP. Triplicate flasks of acid-inhibited controls (pH ≤ 2) containing unlabeled PHE were prepared by adding ca. 0.7 ml of 85% phosphoric acid. All flasks were incubated on an orbital shaker (250 r.p.m.; 21°C) in the dark. At the endpoint of each SIP incubation – defined as the time when the extent of mineralization of the ^14^C-labeled substrate began to approach an asymptote – whole DNA from the total volume in the duplicate flasks amended with the [U-^13^C]-PHE and the corresponding duplicate set with unlabeled PHE was extracted as previously described (Tillett and Neilan, 2000).

### Caesium Chloride (CsCl) Gradient Ultracentrifugation and Identification of ^13^C-Enriched DNA

To separate ^13^C-enriched and unenriched DNA, total extracted DNA from each sample was added to caesium chloride (CsCl) solutions (1.72 g ml^-1^) for isopycnic ultracentrifugation and gradient fractionation, as previously described (Jones et al., 2011). Five microliters of purified *Escherichia coli* DNA (ca. 40 ng ml^-1^) was added and mixed into each tube as an internal standard of unlabeled DNA prior to ultracentrifugation. Denaturing gradient gel electrophoresis (DGGE) was then performed on each fraction to visualize the separation of DNA. For this, PCR amplification of each fraction was carried out with primers 63f-GC (Marchesi et al., 1998) and 517r (Muyzer et al., 1993) using a PCR program as described by Yu and Morrison (2004). PCR products were confirmed on a 1.5% (w/v) agarose gel alongside a HindIII DNA ladder (Invitrogen, Carlsbad, CA, USA). DGGE was performed using 6.5% acrylamide gels containing a denaturant range of 30–60% (100% denaturant contains 7.0 M urea and 40% molecular-grade formamide). After electrophoresis for 16 h at 60°C and 60 V, gels were stained with ethidium bromide at 1:25 000 dilutions for 15 min. Gel image colors were inverted, adjusted for contrast, and cropped to only the regions displaying bands with the GNU Image Manipulation Program (GIMP; version 2.6.8).

### 16S rRNA Gene Libraries of ^13^C-Enriched DNA

To identify PHE-degrading bacteria, a 16S rRNA gene clone library comprising 96 clones was prepared from the ^13^C-enriched DNA fractions (Singleton et al., 2006) using general bacterial primers 27f and 1492r for PCR amplification, followed by partial sequencing with primer 27f (Wilmotte et al., 1993) at the Beckman Coulter Genomics sequencing facility (Danvers, MA, USA). The ^13^C-enriched heavy DNA fractions were selected based on the DGGE evidence, which is discussed below. After excluding vector sequences, poor-quality reads and chimeras, the clone sequences were grouped into operational taxonomic units (OTUs) based on applying a 97% sequence identity cutoff. Using the complete linkage clustering and dereplicate tools available at the Pyrosequencing Pipeline tool of RDP-II (Cole et al., 2009), representative sequences were selected to represent dominant OTUs identified in each of the libraries. Near-complete 16S rRNA gene sequences for the represented sequences were obtained at the University of North Carolina at Chapel Hill Genome Analysis Facility. Sequences were edited and assembled using the program Sequencher 4.8 (Gene Codes Corp., Ann Arbor, MI, USA). The BLASTN search program and RDP-II (Maidak et al., 1999) were used to check for close relatives and phylogenetic affiliation.

### Real-Time Quantitative PCR

To quantify genes of the most dominant OTU, primers for real-time quantitative PCR (qPCR) were developed using the Probe Design and Probe Match tools of ARB, as previously described (Gutierrez et al., 2011). Primer specificity was confirmed with the Probe Check tool of RDP-II. The optimal annealing temperature of each primer pair was determined using an Eppendorf (Hauppauge, NY, USA) Mastercycler gradient thermal cycler. The template for these reactions, and for the construction of respective standard curves for quantitative PCR, was a plasmid containing a representative sequence that had been linearized using PstI (New England BioLabs, Ipswich, MA, USA) and purified using the QIAquick nucleotide removal kit (Qiagen, Valencia, CA, USA). The qPCR primer pairs, their amplification efficiency (Pfaﬄ, 2001), optimal annealing temperature, detection limit and RDP hits are shown in **Table 1**. To confirm the fractions from the DGGE profiles that corresponded to unlabeled DNA, the abundance of the *E. coli* 16S rRNA genes was quantified in each fraction using *E. coli* primers ECP79f (5′-GAAGCTTGCTTCTTTGCT-3′) and ECR620r (5′-GAGCCCGGGGATTTCACA-3′) and a qPCR program with an annealing temperature of 55°C and an extended extension step of 45 s (Sabat et al., 2000).

**Table 1:** Quantitative PCR primers developed and used in this study.

Target OTU	Primer name	Primer sequence (5′→3′)	T_m_ (°C)^a^	qPCR standard^b^	Amplicon length	Amplification efficiency^c^	Detection limit^d^	RDP hits^e^
1	Cyc-467f	AACCTTAGGCCCTGACGT	57	Phenanthrene 1	128	1.68	21	81
	Cyc-577r	TGTTTAACCGCCTACGCG						83 (68)

*^a^Empirically determined PCR annealing temperature.*

*^b^Representative clone sequence from which plasmid DNA was used to generate a standard curve. The plasmid was linearized with PstI.*

*^c^Amplification efficiency (Pfaﬄ, 2001) with operational taxonomic unit (OTU)-specific primers.*

*^d^Detection limit of the qPCR assay expressed as number of 16S rRNA gene copies per milliliter of culture.*

*^e^Number of sequences returned by the Ribosomal Database Project II release 10.18 (Cole et al., 2009; excluding sequences from this study) with no mismatches to primer pairs. Value in parentheses is the total hits that the primer pair targets.*

Purified DNA from time-series incubations with unlabeled hydrocarbon was quantified using a NanoDrop ND-3300 fluorospectrometer (Thermo, Waltham, MA, USA) and the Quant-iT Picogreen double-stranded DNA (dsDNA) kit (Invitrogen). As duplicates of the separated ^12^C- and ^13^C-labeled incubations for each of the three SIP incubations displayed similar distributions of DNA in the fractions, as well as similar DGGE profiles, only the replicate incubation whose fractions contained the highest total amount of DNA was used for further analyses. SIP-identified sequences were quantified in each separated SIP fraction using triplicate reactions by qPCR, as described previously (Singleton et al., 2006). Single reactions were performed on DNA extracted from each of the triplicate samples from the time series incubations containing unlabeled hydrocarbon.

### Isolation of Phenanthrene-Degrading Strains and their Mineralization of ^14^C Phenanthrene

The oil-contaminated surface (0–4 cm) core samples (4571-2 and 4567-24) from the Guaymas Basin were used to isolate bacteria capable of degrading polycyclic aromatic hydrocarbons (PAHs). For this, PHE was used as a representative growth substrate for PAH-degrading organisms. Samples (5 μl) of sediment were streaked directly onto ONR7a agar plates that were then sprayed with PHE dissolved in acetone (ca. 5% w/v) as the sole source of carbon and energy (Kiyohara et al., 1982). The acetone volatilizes off immediately in the process of spraying, leaving behind a thin layer of the PHE on the agar surface. Agar plates were stored in closed plastic bags in the dark at room temperature for up to 4 weeks. Colonies forming clearing zones were picked and subcultured onto fresh ONR7a agar medium amended with the PHE until pure cultures were obtained prior to storage in glycerol (30% v/v) at -80°C.

The potential of the strains to mineralize ^14^C-labeled PHE was determined as described above. For preparation of inocula for these experiments, each strain was grown in ONR7a liquid medium amended with Na-pyruvate (0.1% w/v) and the cell biomass washed several times with fresh ONR7a prior to use.

### 16S rRNA Gene Sequencing and Phylogenetic Analysis

Total genomic DNA of isolated strains was recovered using the method of Tillett and Neilan (2000). The 16S rRNA genes were amplified by PCR with primers 27f (Wilmotte et al., 1993) and 1492r (Lane, 1991). The resulting product was then cloned into the plasmid PCR4-TOPO using the TOPO-TA cloning kit for sequencing (Invitrogen, Carlsbad, CA, USA). The insert was sequenced with primers M13f, M13r, 338f, and 338r (Amann et al., 1990); 907f (Lane et al., 1985); and 907r (Wilmotte et al., 1993) at the University of North Carolina Genome Analysis Facility. Sequences were assembled using the program Sequencher 4.8 (Gene Codes Corp., Ann Arbor, MI, USA). The consensus sequence was submitted to GenBank and checked for close relatives and phylogenetic affiliation using the BLAST search program and RDP-II (Maidak et al., 1999). The search results were used as a guide for tree construction. Additional related 16S rRNA sequences identified from the BLASTN and RDP-II search were retrieved from GenBank.

The 16S rRNA sequences of the isolated strains and SIP-identified sequence were aligned using CLUSTAL_X (Thompson et al., 1994) with the identified close relatives. A neighbor-joining tree was constructed with bootstrapped replication (1000 times) and *Zymobacter palmae* (D14555) was used as an outgroup.

### Nucleotide Sequence Accession Numbers

The 16S rRNA gene sequence of *Cycloclasticus* SIP clone PHE1 and the isolated strains *Cycloclasticus* sp. strain BG-2, *Halomonas* sp. strain BG-3a, *Thalassospira* sp. strain BG-3b, and *Lutibacterium* sp. strain BG-4 were deposited with GenBank under accession numbers KF875697, KF875699, KM404161, KM404162, and KM404163, respectively.

## Results and Discussion

### Exposure of Sediment Samples to Labeled and Unlabeled PAHs

**FIGURE 2:**
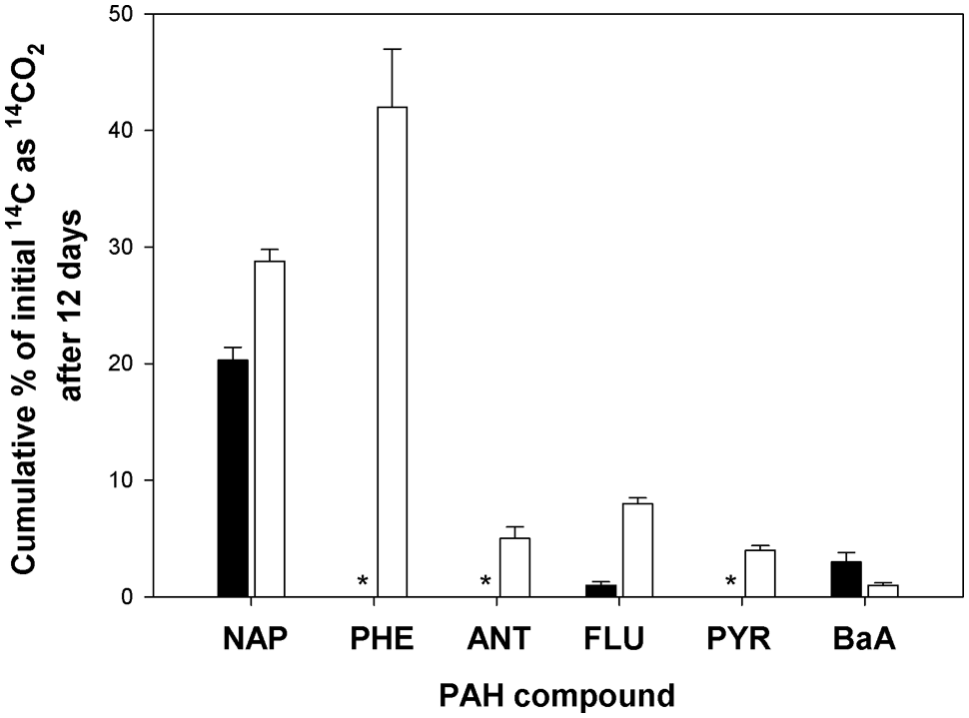
**Cumulative ^14^CO_2_ recovered from incubations with [^14^C]-naphthalene (NAP), phenanthrene (PHE), anthracene (ANT), fluoranthene (FLU), pyrene (PYR), or benz[*a*]anthracene (B*a*A) by surface (0–4 cm) sediment cores 4571-2 (solid bar) and 4567-24 (open bar) during incubation at 21°C for 12 days, as compared to acid-killed controls.** Bars are the averages and SD from triplicate incubations. *values for cumulative ^14^CO_2_ recovered were equal or below that of the respective control incubations.

We determined the potential of the bacterial community in the two surface sediment core samples (4571-2 and 4567-24) to mineralize various ^14^C-labeled PAHs (NAP, PHE, ANT, FLU, PYR, or BaA), since these hydrocarbons have been shown to be present in oily surficial sediment samples at Guaymas (Bazylinski et al., 1988). This was important to thereby inform our choice of the hydrocarbon(s) that would be most suitable for obtaining sufficient incorporation of the ^13^C into biomass, including DNA, since mineralization of a substrate can be suggestive of growth on that substrate. ^14^C-hydrocarbon incubations conducted at 4°C with each of the six hydrocarbons and the two sediment samples yielded very low levels of mineralization (<0.5% mineralized of total hydrocarbon; data not shown). As shown in **Figure 2**, ^14^C incubations at 21°C using the 4571-2 sediment as inoculum revealed that significant levels of NAP had been mineralized (20.3 ± 1.1% cumulative ^14^CO_2_ captured of total initial ^14^C), whereas low mineralization levels (<3.5% cumulative ^14^CO_2_ captured of total initial ^14^C) were measured for FLU and BaA. PHE, ANT, and PYR were not mineralized by the bacterial community in the 4571-2 sediment sample. Conversely, all six of these PAHs were mineralized by the 4567-24 sediment sample at 21°C, with highest levels of cumulative ^14^CO_2_ captured from PHE (42.0 ± 7.0%) and NAP (28.8 ± 1.0%) of total initial ^14^C for each of these compounds. Whilst the oil-rich core 4571-2 was expected to have yielded higher mineralization levels than the quite oxidized 4567-24 sediment core, the converse which was measured may be attributed to the microbial community of core 4567-24 having been more amenable to aerobic conditions than the sulfide-adapted microbial inhabitants of core 4571-2. Cultured strains and uncultured clones of aromatic-degrading sulfate reducing bacteria, mainly belonging to the *Desulfobacteraceae*, have been described from Guaymas sediments (Phelps et al., 1998; Kniemeyer et al., 2003), and most likely play a significant role in the anaerobic oxidation and complete degradation of aromatic hydrocarbons at Guaymas. For SIP, achieving sufficient incorporation of the labeled carbon under short incubation times is desirable in order to minimize the potential for cross-feeding (see below). Hence, based on the significant mineralization of PHE measured with the 4567-24 sediment sample at 21°C, SIP experiments were subsequently conducted using this PAH, sediment sample and incubation temperature.

**FIGURE 3:**
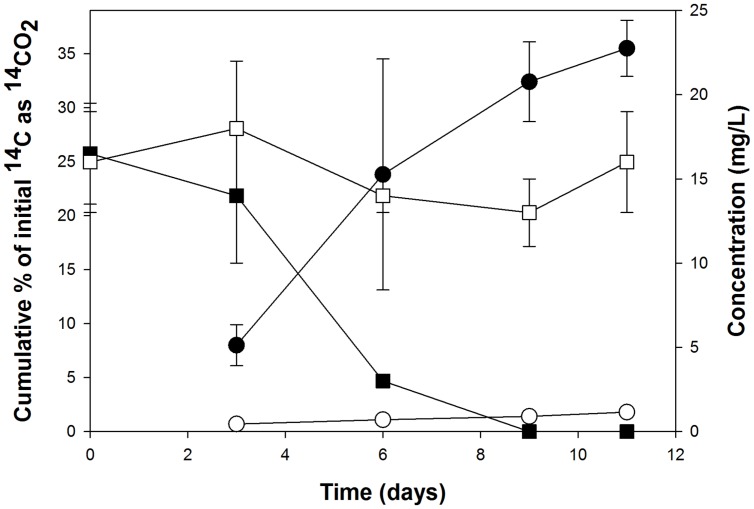
**Cumulative ^14^CO_2_ recovered from the incubations with ^14^C-labeled PHE (circles) that were run in parallel to the stable-isotope probing (SIP) incubations, and the respective removal of this polycyclic aromatic hydrocarbon (PAH) in incubations with the corresponding unlabeled substrates as measured by HPLC (squares).** Each data point is the mean of results from triplicate flasks ± SD. Filled symbols represent live (uninhibited) cultures; open symbols represent acid-inhibited controls. Some error bars are smaller than the symbol.

Careful attention must be employed in the design and execution of SIP in order for it to yield interpretable, unambiguous results. One of the main challenges in SIP is obtaining sufficient incorporation of the ^13^C into biomass, which in the case for DNA-SIP, its enrichment into DNA. Whilst the extent of labeling can be increased with longer incubation times, this can lead to the ^13^C becoming distributed among other members of the microbial community – i.e., those not necessarily directly capable of metabolizing the isotopically labeled substrate – by cross-feeding on ^13^C-labeled metabolic byproducts, intermediates, or dead cells (Lueders et al., 2004). To avert this, we had set up several ^12^C and ^14^C incubations that ran in parallel to the ^13^C incubations in order to tractably measure the degradation (by GC-MS) and mineralization (by scintillation counting) of the PHE to help guide our selection of the point at which to terminate the ^13^C incubations (endpoint of experiment) whereby sufficient ^13^C incorporation had been achieved with minimal cross-feeding. As shown in **Figure 3**, complete removal of the PHE occurred after day 9, whereas mineralization of the ^14^C substrate appeared to reach an asymptote by day 11. Based on these results, the endpoint selected for extraction of DNA from ^13^C incubations was 11 days. DNA extractions were performed on each of the duplicate ^13^C incubations for subsequent isopycnic ultracentrifugation to isolate the ^13^C-enriched ‘heavy’ DNA for analysis.

All live (non-acid treated) incubations were observed to produce a rusty-yellowish coloration after 3 days. This is suggestive of the extracellular accumulation of an oxidized intermediate(s) from the metabolism of PHE, as has been observed previously for the accumulation of 1-hydroxy-2-naphthoate during PHE degradation (Stringfellow and Aitken, 1994).

### Identification of ^13^C-Labeled 16S rRNA Genes

**FIGURE 4:**
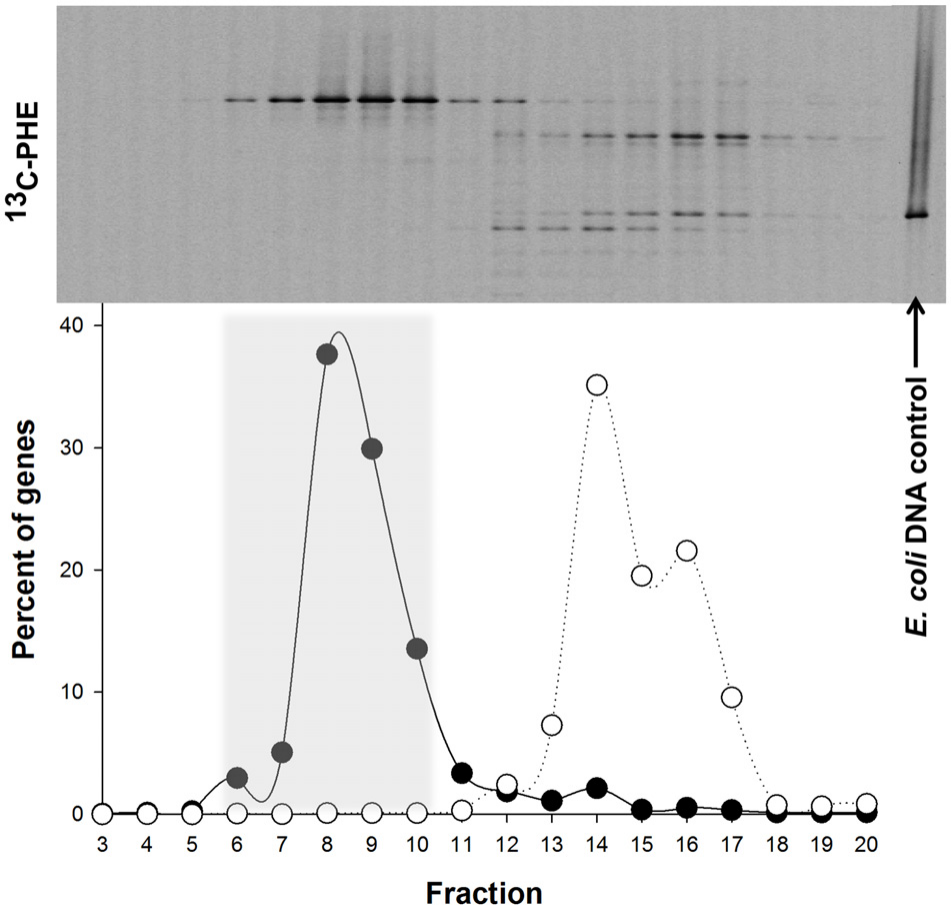
**Distribution of the ‘heavy’ and ‘light’ DNA in separated SIP fractions.** The top of the panel shows the denaturing gradient gel electrophoresis (DGGE) profiles of bacterial PCR products from separated [^13^C]-PHE fractions with decreasing densities from left to right. The position of unlabeled *Escherichia coli* DNA, which was used as an internal control in all three isopycnic centrifugations, is shown on the right. The distribution of qPCR-quantified 16S rRNA gene sequences in fractions from [^13^C]-PHE incubations is shown below the DGGE image for *Cycloclasticus* (●) and for *E. coli* (○). Fractions 6–10 (shaded area) were determined to represent ^13^C heavy DNA and were combined for further analysis. Gene abundance in a fraction are presented as a percentage of the total bacterial 16S rRNA genes quantified in the displayed range of fractions. DGGE banding patterns for a given fraction are aligned with the corresponding gene abundance data below.

Denaturing gradient gel electrophoresis analysis of the fractions derived from the labeled and unlabeled incubations showed clear evidence of isotopic enrichment of DNA in ^13^C-PHE incubations, separation of ^13^C-labeled and unlabeled DNA, and different banding patterns between the ^13^C-enriched and unenriched DNA fractions (**Figure 4**). One band in particular was especially dominant in fractions containing ^13^C-enriched DNA. For the ^13^C-incubation shown in **Figure 4**, fractions 6–10 were combined and used in the generation of the 16S rRNA gene clone library. Fractions 4–8 of the duplicate gradient were combined in a similar fashion (data not shown). After excluding vector sequences, poor sequence reads, chimeras, and singleton sequences, the clone library constructed from pooled ^13^C-enriched DNA comprised 68 sequences. Of these 68 sequences, 3 OTUs were identified of which two were singleton sequences affiliated to *Marinobacterium* and *Propionibacterium* and not further analyzed. OTU-1, designated SIP clone PHE1, which comprised the majority (95%) of the 68 sequences (>99% sequence identity), was found affiliated to the genus *Cycloclasticus*.

**FIGURE 5:**
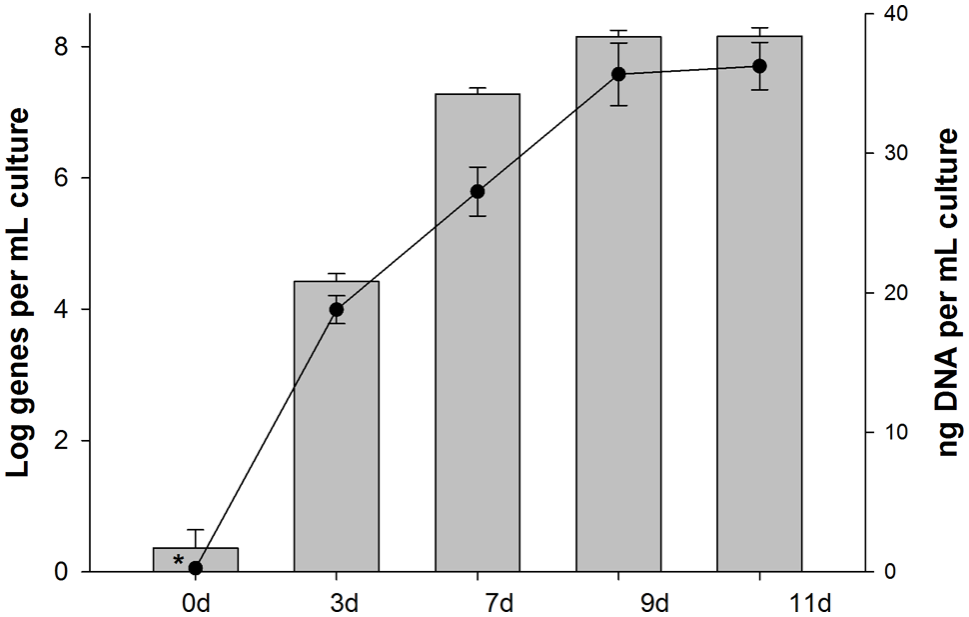
**Abundance of 16S rRNA genes of *Cycloclasticus* (SIP clone PHE1) in samples collected from incubations with unlabeled PHE that were run in parallel during SIP.** Bars are the averages and SD of results from triplicate qPCRs measuring the abundance of group-specific 16S rRNA genes. Circles are the means and standard deviations of triplicate measurements of the total mass of DNA per sample. Bars or data points with asterisks represent values with one or more readings below the quantification limit of the assay and are presented as the largest possible value for that point.

During incubations of the 4567-24 sediment sample with unlabeled PHE in parallel with the SIP incubation, the abundance of 16S rRNA genes for SIP clone PHE1 (OTU-1) increased by several orders of magnitude (**Figure 5**), thus providing further confirmation of its enrichment on PHE as a growth substrate. By day 3 of the PHE enrichment, the gene abundance increased by over three orders of magnitude, coinciding with the time-frame for the initial stages of disappearance, and mineralization of this compound (**Figure 3**). By day 9, the gene abundance had increased by ca. six orders of magnitude, coinciding with the almost complete disappearance and high mineralization rate of the PHE. The increase in gene abundance coincided with an increase in the total concentration of DNA, an indicator of cell growth. Collectively, the low bacterial diversity identified in the heavy DNA fractions, which is almost exclusively represented by the *Cycloclasticus* OTU (SIP clone PHE1), and the dramatic increase in the abundance of these organisms in the incubations with unlabeled PHE, strongly supports that this OTU was solely responsible for degradation of the PAH. In addition, since growth of these organisms coincided with PAH disappearance and the appearance of their 16S rRNA genes only in the most heavily enriched ^13^C-DNA fractions of incubations containing the labeled substrate, their presence in clone libraries was unlikely due to cross-feeding on a PAH metabolite. We cannot, however, disregard the possibility that other bacterial taxa in the Guaymas 4567-24 sediment sample also possessed the capacity to degrade PHE or its metabolites – they were merely not strongly represented in the most highly ^13^C-enriched fractions analyzed. However, as discussed below, we also isolated PHE-degrading strains from this sediment sample that are affiliated to other genera. In previous pyrosequencing analyses of the bacterial diversity of Guaymas Basin sediments, *Cycloclasticus* related sequences composed 0.12% of the average bacterial community at nearby sites, suggesting that representatives of the clade may be poised to act in this oily habitat (Biddle et al., 2012).

Most studies that have reported the identification of *Cycloclasticus* in marine sediments have been conducted in shallow waters. Using PCR-DGGE and 16S rRNA analysis, Cui et al. (2008) identified a *Cycloclasticus* ribotype in a deep sub-surface sediment core collected at 3542 m depth from the mid Atlantic Ridge and posited these organisms to play a key role in PHE degradation. However, since the authors were unable to isolate a representative of these organisms, likely owing to their poor cultivability, this precluded the opportunity to directly determine the potential of these organisms to degrade PAHs. In a study by Goetz and Jannasch (1993), a number of aerobic obligate aromatic-degrading bacteria were isolated from Guaymas sediment that, although not phylogenetically characterized by 16S rRNA sequencing, represent good candidates as possible members of the genus *Cycloclasticus*. Our use of DNA-SIP with a ^13^C-labeled PAH demonstrates the power of this molecular tool in uncovering the diversity of PAH-degraders in deep-sea sediment where they may be a minority portion of the overall community, but that may contribute significantly to the degradation of oil hydrocarbons.

### Isolation and Molecular Analysis of PAH-Degrading Strains

Polycyclic aromatic hydrocarbon-degrading bacteria were isolated from surface (0–4 cm) sediment core sample 4567-24 with enrichment on PHE-sprayed agar plates. Several colonies surrounded by clearing zones grew out on the plates, which is indicative of their capacity to utilize the PHE as a sole source of carbon and energy. Subsequent purification and sequencing identified four morphologically distinct isolates designated strains BG-2, BG-3a, BG-3b, and BG-4. Phylogenetic analysis based on 16S rRNA gene sequences indicated that the strains, respectively, belong to the genus *Cycloclasticus*, *Halomonas*, *Thalassospira*, and *Lutibacterium*. Members comprising each of these genera have been described with PAH-degrading qualities, whilst members of *Cycloclasticus* are recognized as obligate degraders of PAHs (Yakimov et al., 2007). They have been commonly found in marine coastal sediments (Dyksterhouse et al., 1995; Geiselbrecht et al., 1998), in surface sediment of the west Pacific (Wang et al., 2008) and, as noted above, in deep sediment cores of the mid Atlantic Ridge (Cui et al., 2008). Strains of *Halomonas* and *Thalassospira* were enriched with crude oil and individual species and mixtures of PAH substrates from the mid Atlantic Ridge (Cui et al., 2008). Though the ability of these four isolates to directly utilize hydrocarbons as a sole source of carbon and energy was not evaluated, their occurrence in deep-sea sediments and enrichment on oil hydrocarbons suggests that these organisms may play a dominant role in the degradation of oil hydrocarbons in deep-sea surficial sediments.

**FIGURE 6:**
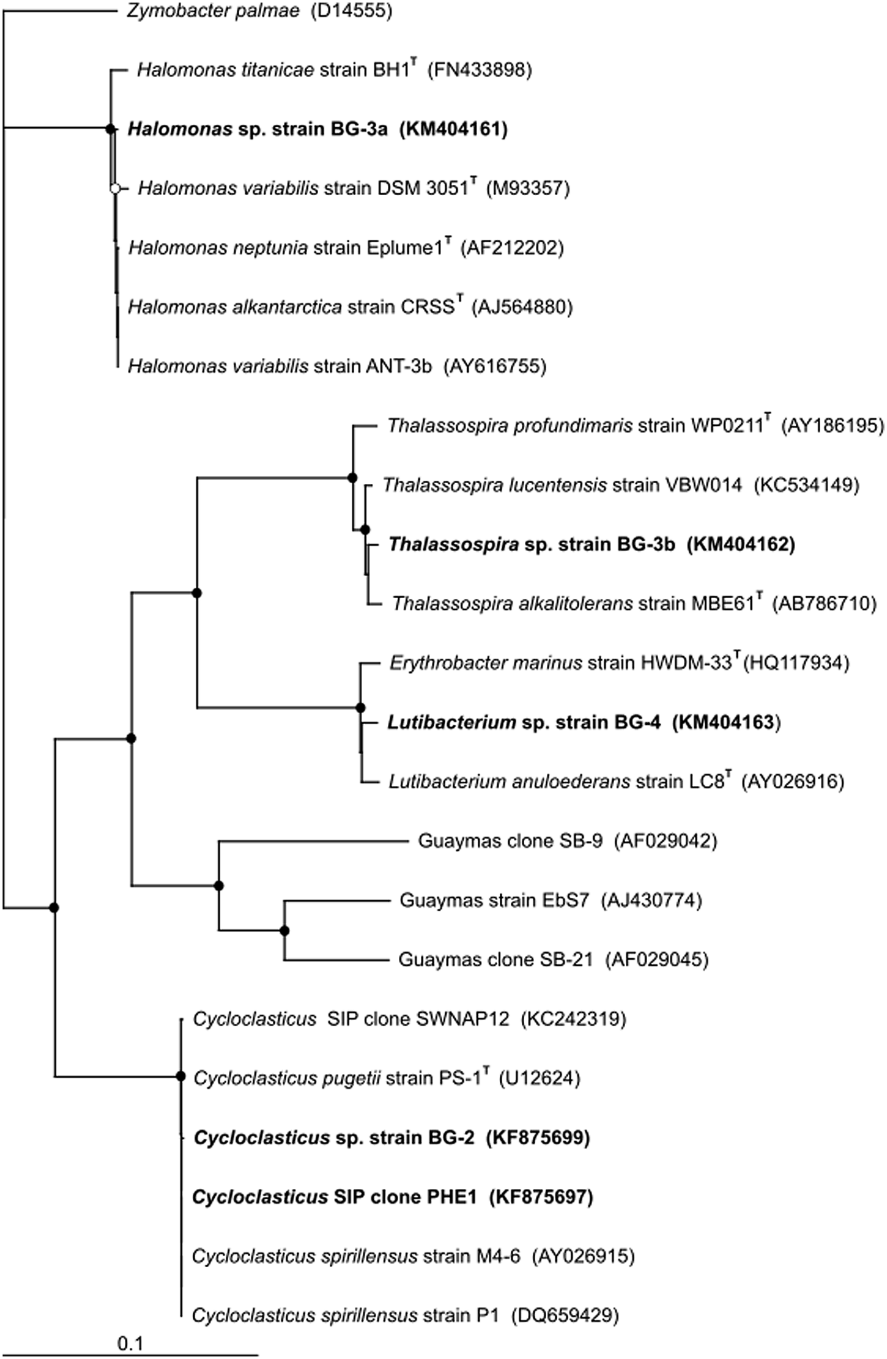
**Phylogenetic tree of SIP clone PHE1 and of the isolated strains from Guaymas Basin surface (0–4 cm) sediment shown alongside closely related sequences and type strains from GenBank**. Guaymas strain EbS7 and Guaymas clones SB-9 and SB-21 are included as representatives of sulfate-reducing bacteria identified at Guaymas Basin that are capable of oxidizing aromatic hydrocarbons. Aerobic PAH-degrading *Cycloclasticus* P1, isolated from deep-sea sediments of the West Pacific, and other strains of this genus isolated from shallower sediment locations (strains M4-6 and PS-1^T^), are also included. The tree was constructed using the neighbor-joining algorithm. Nodes with bootstrap support (1000 replications) of at least 65% (○) and 90% (●) are marked. Accession numbers of all sequences are given in parentheses. The scale bar indicates the difference of number of substitutions per site.

The near-complete 16S rRNA gene sequence (>1400 bp) of each of these PHE-degrading strains were compared with related GenBank sequences, including sequences from studies investigating hydrocarbon-degrading bacteria in deep-sea sediments (**Figure 6**). From a BLAST analysis, the highest level (99.9%) of sequence identity for strain BG-2 was to *Cycloclasticus* sp. strain P1 isolated from deep-sea sediment of the West Pacific at 2682 m water depth (Wang et al., 2008), *Cycloclasticus spirillensus* strain M4-6 isolated from marine macrofaunal burrow sediments of Lowes Cove in Maine, USA (Chung and King, 2001), and to *Cycloclasticus* sp. clone SWNAP12 which was identified in ^13^C-enriched DNA of a SIP enrichment of a surface oil slick sample collected during the Deepwater Horizon oil spill (Gutierrez et al., 2013b). The next closest cultivated relative to BG-2 was *C. pugetii* strain PS-1^T^ (99.7% sequence identity) isolated from marine sediment of Puget Sound (Dyksterhouse et al., 1995). Notably, isolated strain BG-2 shared 99.7% 16S rRNA gene sequence identity with SIP clone PHE1. Strain BG-3a was most closely related to the type strains *Halomonas alkaliantarctica* strain CRSS^T^ (Poli et al., 2007; 99.4% sequence identity), *H. neptunia* strain Eplume1^T^ (Kaye et al., 2004; 99.3% sequence identity), and the exopolysaccharide (EPS)-producer *H. variabilis* strain ANT-3b (Pepi et al., 2005; 99.3% sequence identity). The next closest cultivated relatives to BG-3a were *H. titanicae* strain BH1^T^ (Sanchez-Porro et al., 2010) and *H. variabilis* strain DSM 3051^T^ (Arahal et al., 2002; 98.8% sequence identity). Strain BG-3b shared 99.4% sequence identity to *Thalassospira lucentensis* strain VBW014 (Rajasabapathy et al., 2014), and to the type strains *T. alkalitolerans* strain MBE#61^T^ (Tsubouchi et al., 2014) and *T. profundimaris* strain WP0211^T^ (Liu et al., 2007) with 99.1% and 99.3% sequence identity, respectively. Strain BG-4 was most closely related to the type strains *Lutibacterium anuloederans* strain LC8^T^ (Chung and King, 2001) and *Erythrobacter marinus* strain HWDM-33^T^ (Jung et al., 2012) with 98.7 and 98.6% sequence identity, respectively. In a previous deep sequencing survey of bacterial diversity at Guaymas basin, *Halomonas* sp. accounted for 0.1% of sequences, whereas *Thalassospira* and *Lutibacterium* were not detected (Biddle et al., 2012). This highlights the importance of using enrichment [cultivation-independent (DNA-SIP) and/or cultivation-dependent] methods to uncover minority taxa that may not be identified by solely sequencing surveys, and to linking these organisms to a particular function – in this case the degradation of hydrocarbons.

Whilst members of the genus *Cycloclasticus* are recognized as obligate degraders of PAHs (Yakimov et al., 2007), only some members comprising the genera *Halomonas*, *Thalassospira*, and *Lutibacterium* have been reported as non-obligate degraders of PAHs (Chung and King, 2001; Melcher et al., 2002; Kodama et al., 2008; Zhao et al., 2010; Gutierrez et al., 2013a). The ability of these isolated strains (BG-2, BG-3a, BG-3b, BG-4) to grow on and degrade PHE was confirmed on ONR7a liquid medium amended with the PAH as the sole carbon source (data not shown). The ability of the strains to mineralize PHE was assessed by measuring for the liberation of ^14^CO_2_ from ^14^C-labeled PHE. After 12 days of incubation, significant levels (*P* < 0.05) of the PAH was mineralized by strain BG-2 (11.7 ± 0.7%), strain BG-3a (5.1 ± 2.3%), strain BG-3b (8.4 ± 3.5%), and strain BG-4 (16.7 ± 4.0%) of total added ^14^C-labeled compound when compared to respective acid-killed controls (data not shown). During these mineralization experiments, a notable feature observed with *Cycloclasticus* sp. strain BG-2 was the production of a rusty-yellow coloration in the medium after a few days, as similarly observed in the SIP incubations (see above).

To the best of our knowledge, this study represents the first application of SIP to identify bacterial taxa with the ability to degrade PAHs in deep-sea sediments. In particular, this work has added to our current knowledge on the diversity of hydrocarbon-degrading bacteria in Guaymas Basin sediments – an environment with abundant sediment surface hydrocarbons (Goetz and Jannasch, 1993). In summary, the dominant representation of *Cycloclasticus* in the ^13^C-enriched clone library and recognition of members in this genus as obligate degraders of PAHs (Yakimov et al., 2007) suggests that *Cycloclasticus* may play an important role in the degradation of these compounds at Guaymas Basin. Using DNA-SIP and cultivation-based methods, we have shown that these organisms and other hydrocarbon-degraders (*Halomonas*, *Thalassospira*, and *Lutibacterium*) co-exist in Guaymas surface sediments. We posit that the activities of these organisms are likely to have a significant influence on the natural attenuation of PAHs and other hydrocarbon constituents of the crude oil that is a natural feature to this deep-sea vent site.

## Conflict of Interest Statement

The authors declare that the research was conducted in the absence of any commercial or financial relationships that could be construed as a potential conflict of interest.

## References

[B1] Aitken M. M. Jones D. M. Larter S. R. (2004). Anaerobic hydrocarbon biodegradation in deep subsurface oil reservoirs. *Nature* 431 291–294. 10.1038/nature02922 15372028

[B2] Amann R. I. Binder B. J. Olson R. J. Chisholm S. W. Devereux R. Stahl D. A. (1990). Combination of 16S rRNA-targeted oligonucleotide probes with flow cytometry for analyzing mixed microbial populations. *Appl. Environ. Microbiol.* 56 1919–1925. 2200342 10.1128/aem.56.6.1919-1925.1990 PMC184531

[B3] Arahal D. R. Ludwig W. Schleifer K. H. Ventosa A. (2002). Phylogeny of the family Halomonadaceae based on 23S and 16S rDNA sequence analyses. *Int. J. Syst. Evol. Microbiol.* 52 241–249. 11837309 10.1099/00207713-52-1-241

[B4] Bazylinski D. A. Farrington J. W. Jannasch H. W. (1988). Hydrocarbons in surface sediments from a Guaymas Basin hydrothermal vent site. *Org. Geochem.* 12 547–558. 10.1016/0146-6380(88)90146-5

[B5] Bennett B. Adams J. J. Gray N. D. Sherry A. Oldenburg T. B. P. Huang H. (2013). The controls on the composition of biodegraded oils in the deep subsurface – Part 3. The impact of microorganisms distribution on petroleum geochemical gradients in biodegraded petroleum reservoirs. *Org. Geochem.* 56 94–105. 10.1016/j.orggeochem.2012.12.011

[B6] Biddle J. F. Cardman Z. Mendlovitz H. Albert D. B. Lloyd K. G. Boetius A. (2012). Anaerobic oxidation of methane at different temperature regimes in Guaymas Basin hydrothermal sediments. *ISME J.* 6 1018–1031. 10.1038/ismej.2011.164 22094346 PMC3329104

[B7] Chanton J. Zhao T. Rosenheim B. E. Joye S. Bosman S. Brunner C. (2015). Using natural abundance radiocarbon to trace the flux of petrocarbon to the seafloor following the Deepwater Horizon oil spill. *Environ. Sci. Technol.* 49 847–854. 10.1021/es5046524 25494527

[B8] Chung W. K. King G. M. (2001). Isolation, characterization, and polyaromatic hydrocarbon degradation potential of aerobic bacteria from marine macrofaunal burrow sediments and description of *Lutibacterium anuloederans* gen. nov., sp. nov., and *Cycloclasticus spirillensus* sp. nov. *Appl. Environ. Microbiol.* 67 5585–5592. 10.1128/AEM.67.12.5585-5592.2001 11722910 PMC93347

[B9] Cole J. R. Wang Q. Cardenas E. Fish J. Chai B. Farris R. J. (2009). The Ribosomal Database Project: improved alignments and new tools for rRNA analysis. *Nucleic Acids Res.* 37 D141–D145. 10.1093/nar/gkn879 19004872 PMC2686447

[B10] Cui Z. Lai Q. Dong C. Shao Z. (2008). Biodiversity of polycyclic aromatic hydrocarbon-degrading bacteria from deep sea sediments of the Middle Atlantic Ridge. *Environ. Microbiol.* 10 2138–2149. 10.1111/j.1462-2920.2008.01637.x 18445026 PMC2702504

[B11] Dumont M. Murrell J. (2005). Stable isotope probing – linking microbial identity to function. *Nat. Rev. Microbiol.* 3 499–504. 10.1038/nrmicro1162 15886694

[B12] Dyksterhouse S. E. Gray J. P. Herwig R. P. Cano Lara J. Staley J. T. (1995). *Cycloclasticus pugetii* gen. nov., sp.nov., an aromatic hydrocarbon-degrading bacterium from marine sediments. *Int. J. Syst. Bacteriol.* 45 116–123. 10.1099/00207713-45-1-116 7857792

[B13] Geiselbrecht A. D. Hedlund B. P. Tichi M. A. Staley J. T. (1998). Isolation of marine polycyclic aromatic hydrocarbon (PAH)-degrading *Cycloclasticus* strains from the Gulf of Mexico and comparison of their PAH degradation ability with that of Puget Sound *Cycloclasticus* strains. *Appl. Environ. Microbiol.* 64 4703–4710. 9835552 10.1128/aem.64.12.4703-4710.1998 PMC90912

[B14] Goetz F. E. Jannasch H. W. (1993). Aromatic hydrocarbon-degrading bacteria in the petroleum-rich sediments of Guaymas Basin hydrothermal vent site: Preference for aromatic carboxylic acids. *Geomicrobiol. J.* 11 1–18. 10.1080/01490459309377928

[B15] Gutierrez T. Berry D. Yang T. Mishamandani S. McKay L. Teske A. (2013a). Role of bacterial exopolysaccharides (EPS) in the fate of the oil released during the Deepwater Horizon oil spill. *PLoS ONE* 8:e67717 10.1371/journal.pone.0067717 PMC3694863 23826336

[B16] Gutierrez T. Singleton D. R. Berry D. Yang T. Aitken M. D. Teske A. (2013b). Hydrocarbon-degrading bacteria enriched by the Deepwater Horizon oil spill identified by cultivation and DNA-SIP. *ISME J.* 7 2091–2104. 10.1038/ismej.2013.98 23788333 PMC3806270

[B17] Gutierrez T. Singleton D. R. Aitken M. D. Semple K. T. (2011). Stable isotope probing of an algal bloom to identify uncultivated members of the Rhodobacteraceae associated with low-molecular-weight polycyclic aromatic hydrocarbon degradation. *Appl. Environ. Microbiol.* 77 7856–7860. 10.1128/AEM.06200-11 21926219 PMC3209190

[B18] Head I. M. Jones D. M. Röling W. F. (2006). Marine microorganisms make a meal of oil. *Nat. Rev. Microbiol.* 4 173–182. 10.1038/nrmicro1348 16489346

[B19] Jones D. M. Head I. M. Gray N. D. Adams J. J. Rowan A. K. Aitken C. M. (2008). Crude-oil biodegradation via methanogenesis in subsurface petroleum reservoirs. *Nature* 451 176–180. 10.1038/nature06484 18075503

[B20] Jones M. D. Singleton D. R. Sun W. Aitken M. D. (2011). Multiple DNA extractions coupled with stable-isotope probing of anthracene-degrading bacteria in contaminated soil. *Appl. Environ. Microbiol.* 77 2984–2991. 10.1128/AEM.01942-10 21398486 PMC3126383

[B21] Jung Y.-T. Park S. Oh T.-K. Yoon J.-H. (2012). *Erythrobacter marinus* sp. nov., isolated from seawater. *Int. J. Syst. Evol. Microbiol.* 62 2050–2055. 10.1099/ijs.0.034702-0 22021578

[B22] Kaye J. Z. Márquez M. C. Ventosa A. Baross J. A. (2004). *Halomonas neptunia* sp. nov., *Halomonas sulfidaeris* sp. nov., *Halomonas axialensis* sp. nov. and *Halomonas hydrothermalis* sp. nov.: halophilic bacteria isolated from deep-sea hydrothermal-vent environments. *Int. J. Syst. Evol. Microbiol.* 54 499–511. 10.1099/ijs.0.02799-0 15023967

[B23] Kiyohara H. Nagao K. Yana K. (1982). Rapid screen for bacteria degrading water-insoluble, solid hydrocarbons on agar plates. *Appl. Environ. Microbiol.* 43 454–457. 16345951 10.1128/aem.43.2.454-457.1982 PMC241847

[B24] Kleindienst S. Herbst F.-A. Stagars M. von Netzer F. von Bergen M. Seifert J. (2014). Diverse sulphate-reducing bacteria of the *Desulfosarcina*/*Desulfococcus* clade are the key alkane degraders at marine seeps. *ISME J.* 8 2029–2044. 10.1038/ismej.2014.51 24722631 PMC4184016

[B25] Kniemeyer O. Fischer T. Wilkes H. Glöckner F. O. Widdel F. (2003). Anaerobic degradation of ethylbenzene by a new type of marine sulfate-reducing bacterium. *Appl. Environ. Microbiol.* 69 760–768. 10.1128/AEM.69.2.760-768.2003 12570993 PMC143655

[B26] Kodama Y. Stiknowati L. I. Ueki A. Ueki K. Watanabe K. (2008). *Thalassospira tepidiphila* sp. nov., a polycyclic aromatic hydrocarbon-degrading bacterium isolated from seawater. *Int. J. Syst. Evol. Microbiol.* 58 711–715. 10.1099/ijs.0.65476-0 18319483

[B27] Lane D. J. (1991). “16S/23S rRNA sequencing,” in *Nucleic Acid Sequencing Techniques in Bacterial Systematics*, eds Stackebrandt E. Goodfellow M. (New York, NY: John Wiley & Sons), 115–175.

[B28] Lane D. J. Pace B. Olsen G. J. Stahl D. A. Sogin M. L. Pace N. R. (1985). Rapid determination of 16S ribosomal RNA sequences for phylogenetic analyses. *Proc. Natl. Acad. Sci. U.S.A.* 82 6955–6959. 10.1073/pnas.82.20.6955 2413450 PMC391288

[B29] Lanza-Espino G. Soto L. A. (1999). Sedimentary geochemistry of hydrothermal vents in Guaymas Basin, Gulf of California, Mexico. *Appl. Geo. Chem.* 14 499–510. 10.1016/s0883-2927(98)00064-x

[B30] Liu C. Wu Y. Li L. Ma Y. Shao Z. (2007). *Thalassospira xiamenensis* sp. nov. and *Thalassospira profundimaris* sp. nov. *Int. J. Syst. Evol. Microbiol.* 57 316–320. 10.1099/ijs.0.64544-0 17267971

[B31] Lueders T. Wagner B. Claus P. Friedrich M. (2004). Stable isotope probing of rRNA and DNA reveals a dynamic methylotroph community and trophic interactions with fungi and protozoa in oxic rice field soil. *Environ. Microbiol.* 6 60–72. 10.1046/j.1462-2920.2003.00535.x 14686942

[B32] Maidak B. L. Cole J. R. Parker C. T. Jr Garrity G. M. Larsen N. Li B. (1999). A new version of the RDP (Ribosomal Database Project). *Nucleic Acids Res.* 27 171–173. 10.1093/nar/27.1.171 9847171 PMC148126

[B33] Marchesi J. R. Sato T. Weightman A. J. Martin T. A. Fry J. C. Hiom S. J. (1998). Design and evaluation of useful bacterium-specific PCR primers that amplify genes coding for bacterial 16S rRNA. *Appl. Environ. Microbiol.* 64 795–799. 9464425 10.1128/aem.64.2.795-799.1998 PMC106123

[B34] McKay L. J. MacGregor B. J. Biddle J. F. Mendlovitz H. P. Hoer D. Lipp J. S. (2012). Spatial heterogeneity and underlying geochemistry of phylogenetically diverse orange and white Beggiatoa mats in Guaymas Basin hydrothermal sediments. *Deep-Sea Res. Part 1* 67 21–31. 10.1016/j.dsr.2012.04.011

[B35] Melcher R. J. Apitz S. E. Hemmingsen B. B. (2002). Impact of irradiation and polycyclic aromatic hydrocarbon spiking on microbial populations in marine sediment for future aging and biodegradability studies. *Appl. Environ. Microbiol.* 68 2858–2868. 10.1128/AEM.68.6.2858-2868.2002 12039743 PMC123915

[B36] Mishamandani S. Gutierrez T. Aitken M. D. (2014). DNA-based stable isotope probing coupled with cultivation methods implicates Methylophaga in hydrocarbon degradation. *Front. Microbiol.* 5:76 10.3389/fmicb.2014.00076 PMC3936186 24578702

[B37] Muyzer G. de Waal E. C. Uitterlinden A. G. (1993). Profiling of complex microbial populations by denaturing gradient gel electrophoresis analysis of polymerase chain reaction-amplified genes coding for 16S rRNA. *Appl. Environ. Microbiol.* 59 695–700. 7683183 10.1128/aem.59.3.695-700.1993 PMC202176

[B38] Orphan V. J. Taylor L. T. Hafenbradl D. DeLong E. F. (2000). Culture-dependent and culture-independent characterization of microbial assemblages associated with high-temperature petroleum reservoirs. *Appl. Environ. Microbiol.* 66 700–711. 10.1128/AEM.66.2.700-711.2000 10653739 PMC91884

[B39] Passow U. Ziervogel K. Asper V. Diercks A.-R. (2012). Marine snow formation in the aftermath of the Deepwater Horizon oil spill in the Gulf of Mexico. *Environ. Res. Lett.* 7:035301; 10.1088/1748-9326/7/3/035301

[B40] Pearson A. Seewald J. S. Eglinton T. I. (2005). Bacterial incorporation of relict carbon in the hydrothermal environment of Guaymas Basin. *Geochim. Cosmochim. Acta* 69 5477–5486. 10.1016/j.gca.2005.07.007

[B41] Pepi M. Cesaro A. Liut G. Baldi F. (2005). An antarctic psychrotrophic bacterium *Halomonas* sp. ANT-3b, growing on n-hexadecane, produces a new emulsifying glycolipid. *FEMS Microbiol. Ecol.* 53 157–166. 10.1016/j.femsec.2004.09.013 16329937

[B42] Peter J. M. Peltonen P. Scott S. D. Simoneit B. R. T. Kawka O. E. (1991). 14C ages of hydrothermal petroleum and carbonate in Guaymas Basin, Gulf of California: implications for oil generation, expulsion, and migration. *Geology* 19 253–256. 10.1130/0091-7613(1991)019<0253:CAOHPA>2.3.CO;2

[B43] Pfaﬄ M. W. (2001). A new mathematical model for relative quantification in real-time RT-PCR. *Nucleic Acids Res.* 29 2002–2007. 10.1093/nar/29.9.e45 PMC55695 11328886

[B44] Phelps C. D. Kerkhof L. J. Young L. Y. (1998). Molecular characterization of a sulfate-reducing consortium which mineralizes benzene. *FEMS Microbiol. Ecol.* 27 269–279. 10.1111/j.1574-6941.1998.tb00543.x

[B45] Poli A. Esposito E. Orlando P. Lama L. Giordano A. de Appolonia F. (2007). *Halomonas alkaliantarctica* sp. nov., isolated from saline lake Cape Russell in Antarctica, an alkalophilic moderately halophilic, exopolysaccharide-producing bacterium. *Syst. Appl. Microbiol.* 30 31–38. 10.1016/j.syapm.2006.03.003 16621401

[B46] Rajasabapathy R. Mohandass C. Colaco A. Dastager S. G. Santos R. S. Meena R. M. (2014). Culturable bacterial phylogeny from a shallow water hydrothermal vent of Espalamaca (Faial, Azores) reveals a variety of novel taxa. *Curr. Sci.* 106 58–69.

[B47] Sabat G. Rose P. Hickey W. J. Harkin J. M. (2000). Selective and sensitive method for PCR amplification of *Escherichia coli* 16S rRNA genes in soil. *Appl. Environ. Microbiol.* 66 844–849. 10.1128/AEM.66.2.844-849.2000 10653763 PMC91908

[B48] Sanchez-Porro C. Kaur B. Mann H. Ventosa A. (2010). *Halomonas titanicae* sp. nov., a halophilic bacterium isolated from the RMS Titanic. *Int. J. Syst. Evol. Microbiol.* 60 2768–2774. 10.1099/ijs.0.020628-0 20061494

[B49] Sauret C. Séverin T. Vétion G. Guigue C. Goutx M. Pujo-Pay M. (2014). ‘Rare biosphere’ bacteria as key phenanthrene degraders in coastal seawaters. *Environ. Pollut.* 194 246–253. 10.1016/j.envpol.2014.07.024 25156140

[B50] Schwarz J. R. Walker J. D. Colwell R. R. (1974a). Deep-sea bacteria: growth and utilization of hydrocarbons at ambient and in situ pressure. *Appl. Microbiol.* 28 982–986. 4451379 10.1128/am.28.6.982-986.1974 PMC186867

[B51] Schwarz J. R. Walker J. D. Colwell R. R. (1974b). Growth of deep-sea bacteria on hydrocarbons at ambient and in situ pressure. *Dev. Ind. Microbiol.* 15 239–249. 10.1128/am.28.6.982-986.1974 PMC186867 4451379

[B52] Singleton D. R. Sangaiah R. Gold A. Ball L. M. Aitken M. D. (2006). Identification and quantification of uncultivated Proteobacteria associated with pyrene degradation in a bioreactor treating PAH-contaminated soil. *Environ. Microbiol.* 8 1736–1745. 10.1111/j.1462-2920.2006.01112.x 16958754

[B53] Stringfellow W. T. Aitken M. D. (1994). Comparative physiology of phenanthrene degradation by two dissimilar pseudomonads isolated from a creosote-contaminated soil. *Can. J. Microbiol.* 40 432–438. 10.1139/m94-071 8050063

[B54] Teske A. Callaghan A. V. LaRowe D. E. (2014). Biosphere frontiers of subsurface life in the sedimented hydrothermal system of Guaymas Basin. *Front. Microbiol.* 5:362 10.3389/fmicb.2014.00362 PMC4117188 25132832

[B55] Thompson J. D. Higgins D. G. Gibson T. J. (1994). CLUSTAL_X: improving the sensitivity of progressive multiple sequence alignment through sequence weighting, position-specific gap penalties and weight matrix choice. *Nucleic Acids Res.* 22 4673–4680. 10.1093/nar/22.22.4673 7984417 PMC308517

[B56] Tillett D. Neilan B. A. (2000). Xanthogenate nucleic acid isolation from cultured and environmental cyanobacteria. *J. Phycol.* 36 251–258. 10.1046/j.1529-8817.2000.99079.x

[B57] Tsubouchi T. Ohta Y. Haga T. Usui K. Shimane Y. Mori K. (2014). *Thalassospira alkalitolerans* sp. nov. and *Thalassospira mesophila* sp. nov., isolated from a decaying bamboo sunken in the marine environment, and description of the genus *Thalassospira*. *Int. J. Syst. Evol. Microbiol.* 64 107–115. 10.1099/ijs.0.056028-0 24021727

[B58] Wang B. Lai Q. Cui Z. Tan T. Shao Z. (2008). A pyrene-degrading consortium from deep-sea sediment of the West Pacific and its key member *Cycloclasticus* sp. P1. *Environ. Microbiol.* 10 1948–1963. 10.1111/j.1462-2920.2008.01611.x 18430013

[B59] Wilmotte A. Van der Auwera G. De Wachter R. (1993). Structure of the 16S ribosomal RNA of the thermophilic cyanobacterium *Chlorogloeopsis* HTF (‘*Mastigocladus laminosus* HTF’) strain PCC7518, and phylogenetic analysis. *FEMS Microbiol. Lett.* 317 96–100. 10.1016/0014-5793(93)81499-P 8428640

[B60] Yakimov M. M. Timmis K. N. Golyshin P. N. (2007). Obligate oil-degrading marine bacteria. *Curr. Opin. Biotechnol.* 18 257–266. 10.1016/j.copbio.2007.04.006 17493798

[B61] Yu Z. Morrison M. (2004). Comparisons of different hypervariable regions of rrs genes for use in fingerprinting of microbial communities by PCR-denaturing gradient gel electrophoresis. *Appl. Environ. Microbiol.* 70 4800–4806. 10.1128/AEM.70.8.4800-4806.2004 15294817 PMC492348

[B62] Zhang Z. Sangaiah R. Gold A. Ball L. M. (2011). Synthesis of uniformly 13C-labeled polycyclic aromatic hydrocarbons. *Org. Biomol. Chem.* 9 5431–5435. 10.1039/c0ob01107j 21670806 PMC10545081

[B63] Zhao B. Wang H. Li R. Mao X. (2010). *Thalassospira xianhensis* sp. nov., a polycyclic aromatic hydrocarbon-degrading marine bacterium. *Int. J. Syst. Evol. Microbiol.* 60 1125–1129. 10.1099/ijs.0.013201-0 19666802

